# An Answer to Dr. F. T. Oates on Treatment of Malarial Hæmatinuria

**Published:** 1894-10

**Authors:** W. W. Walker

**Affiliations:** Schulenburg, Texas


For Texas Medical Journal.

### AN ANSWER TO DR. T. F. OATES ON TREATMENT OF MALARIAL HÆMATINURIA.

BY W. W. WALKER, M. D., SCHULENBURG, TEXAS.

I SHALL not try to enter into a discussion of the ætiology or pathology of malarial hæmatinuria, as I have neither time nor ability, nor am I equipped with microscopic or chemical apparatus for original research. I accept the disease to be of malarial origin; so does Dr. Oates, but he flies off from his base as soon as a chill arrives, accompanied with bloody urine, and claims the climax to be reached "by reason of imperfect oxygenation consequent upon diminished phosphates." How does the doctor know there is "diminished phosphates?" Has he made original researches? If so, he ought to give us the methods by which he arrives at his conclusions. He must depend upon personal research, as none but "parrots" have written on this subject.

My opinion is, that the disease is due to malaria (whatever that may be), first, last, and all the time, and I am one of those who "would drawl out, in parrot-like style, treat with calomel and quinine."

I find I am in good company, as many of the class of 1869 and 1870, in Medical Department University of Louisiana, will recollect Prof. Bemiss' case in ward 18, Charity Hospital. Jerry Cronan, Irish section hand, brought in from Mobile & N. O. R. R., comatose; history of chills and fevers; started out to work with gang, and fell off of hand car. Prof. Bemiss' attention was called to bloody stains on trousers. I drew off about two ounces bloody urine. Diagnosis: "Malarial hematuria," with coma. (The class will recollect when Mr. Monette or Mr. Penney were not present Prof. Bemiss nearly always had me to write his prescriptions).

Patient was ordered sixty grs. calomel, to be followed in four hours by copious enema. Quinine was given in one drachm doses every three hours, until an ounce was given, then the patient was put on:

℞. Quinia sul., ℨi; tinct. ferri per chl., ℨiv; aqua ad. q. s., ℥vi. M. S. Give a tablespoonful every six hours.

In four days this case was brought into amphitheater of hospital and was the basis of a lecture on the subject.

At the request of Drs. Yates, Blakemore and others, Prof. Bemiss published an article on malarial fevers in New Orleans *Medical and Surgical Journal*, of which he was one of the editors. Surely Dr. Oates is not ignorant of the writings of Prof. Joseph Jones on malarial fevers.

Malarial hæmatinuria was very prevalent along the Colorado river and adjacent creeks, from 1867 to 1876. Such noted physicians as Dr. T. M. Blakemore, of Abilene, now living, Drs. Yates, Lewis, Routh, Thomas, Watts and others, all now dead, opposed the use of quinine in malarial hæmaturia, and lost two out of three of their cases. As a young physician, I was afraid to go against their opinions until I had lost my first three cases.

My first case treated with calomel and quinine was September 17th, 1872. J. P. Frierson, farmer, aged 24; residence, Peach creek (father had just died of hæmaturia). I was in the house when the chill came on; it was accompanied with bloody urine. I gave immediately:

℞. Alcohol, ʒiv; tinct. opii. spts. sul. ether aa, ʒss; quinia, grs. x; aqua, q. s. In half an hour chill had subsided. Gave calomel, ʒi; pulvis doveri, grs. x. M. Ft. charts No. iij. S. Give one powder every three hours; and quinia sul., ℈iv; ft. sol. (with aro. sul. acid), and water, q. s. ad., ℥iv. S. Give a tablespoonful every three hours between doses of calomel; in eight hours the calomel moved bowels; the patient vomited a large amount of yellow bile. As soon as vomiting ceased I could see the yellow color fading from the skin, like a light cloud passing under the sun.

The patient is now living, notwithstanding one of my professional brethren brought his fist down on the counter in Cockrill's store, and said, "I'll be damned if Walker don't kill him." My friend and old partner, Dr. J. C. B. Renfro, of Houston, will verify the above.

I gave to a Bohemian, now living near here, in 1873, during the algid stage of malarial hæmatinuria:

℞. Quinia sul., ʒi; alcohol, ℥i; tinct. opii., sul. ether, aa, ʒi; aqua, q. s. S. Take at once; and followed it up with calomel and quinine.

I have lost very few cases since I adopted Prof. Bemiss' line of treatment.

My opinion, based on clinical experience, is,—malarial hæmatinuria bears the same relation to chills and fever a Springfield rifle shot does to a shot from a ten-inch rifle cannon ball; it takes an impediment to stop the rifle ball, and it takes one *in propor-*

*tion* to stop the cannon ball; hence, I shall continue the heroic calomel and quinine treatment until some physician can demonstrate to me a better. I am not prone to wander after new gods or desert old friends, even at the risk of being called a "parrot."

